# The high risk for type 2 diabetes among ethnic minority populations is not explained by low-grade inflammation

**DOI:** 10.1038/s41598-019-56596-4

**Published:** 2019-12-27

**Authors:** Mirthe Muilwijk, Max Nieuwdorp, Marieke B. Snijder, Michel H. P. Hof, Karien Stronks, Irene G. M. van Valkengoed

**Affiliations:** 1 Amsterdam UMC, University of Amsterdam, Department of Public Health, Amsterdam Public Health research institute, Meibergdreef 9, Amsterdam, The Netherlands; 2 Amsterdam UMC, University of Amsterdam, Department of Internal and Vascular Medicine, Meibergdreef 9, Amsterdam, The Netherlands; 3 Amsterdam UMC, University of Amsterdam, Department of Clinical Epidemiology, Biostatistics and Bioinformatics, Meibergdreef 9, Amsterdam, The Netherlands

**Keywords:** Type 2 diabetes, Epidemiology, Chronic inflammation

## Abstract

Our aim was to identify whether low-grade inflammation, reflected by C-reactive protein (CRP), explains the higher risk for incident type 2 diabetes (T2D) among ethnic minorities. We included 837 Dutch, 712 South-Asian Surinamese, 797 African Surinamese, 804 Ghanaian, 817 Turkish and 778 Moroccan origin participants of the HELIUS study (Amsterdam, the Netherlands). We used multiple linear regression to assess ethnic differences in CRP levels. We determined the association of CRP with T2D and the modifying effect of ethnicity by cox regression, and compared hazard ratios for the association between ethnicity and T2D before and after adjustment for CRP. CRP levels were higher in ethnic minority groups than in Dutch origin participants. CRP was associated with a higher T2D incidence, similarly across ethnic groups (overall HR per SD 1.38 [95% CI 1.14; 1.68]). However, the association was attenuated and no longer statistically significant after adjustment for adiposity measures (HR 1.11 [95% CI 0.90; 1.37]). CRP accounted for a very small part of the ethnic differences in T2D, but only in models unadjusted for adiposity. Low-grade inflammation does not substantially contribute to the higher risk of T2D among ethnic minority populations compared to the Dutch.

## Introduction

The incidence of type 2 diabetes (T2D) is 2–12 times higher in South-Asian and African populations compared to European populations^1,2^. The causes of this increased risk have not been fully elucidated, but might be partly explained by higher levels of low-grade inflammation among ethnic minorities. T2D is characterized by a state of low-grade inflammation, which may mainly be attributed to over-nutrition, excess adipose tissue and lipotoxicity^3^. Other sources of inter-individual variability in low-grade inflammation include ageing, genetics and lifestyle factors such as smoking^3–5^. Low-grade inflammation affects both insulin signalling and β-cell function^6^. C-reactive protein (CRP) is a marker of low grade inflammation. If CRP explains part of the higher risk for T2D among ethnic minorities, future studies may investigate whether targeting underlying causes of low-grade inflammation, besides overweight and smoking, may reduce the higher T2D risk among ethnic minorities and the incidence of T2D in general.

CRP has been associated with incidence of T2D in some populations^5,7^. Underlying mechanisms are mainly related to harmful effects of cytokines amongst which direct interference in insulin signal transduction and β-cell deterioration. Associations between CRP and T2D were, however, inconsistent across studies, which may be related to ethnicity^8^. Importantly, the association has not yet been confirmed among high-risk South-Asian, African Surinamese, Ghanaian, Turkish and Moroccan origin populations. If confirmed, and if CRP levels are found/confirmed to be high, low-grade inflammation may explain the increased risk for T2D among ethnic minorities.

Previous studies already showed ethnic differences in CRP levels to exist^9,10^, which may indicate a pro-inflammatory state which makes some groups, e.g. South-Asians, especially vulnerable for T2D^11–13^. CRP levels were for instance shown to be similar among Ghanaians and Dutch^14^, but higher among Moroccans and Turks than among Dutch^15^, and higher among South-Asians than Europeans^12,16,17^.

Our study set out to investigate the contribution of low-grade inflammation to differences in T2D incidence amongst six different ethnic origin groups living in Amsterdam, the Netherlands. First, we describe differences in CRP levels between people of Dutch, Turkish, Moroccan, Ghanaian, South-Asian Surinamese and African Surinamese origin. Second, we investigated the association of CRP with incident T2D. Finally, we determined whether the increased risk for T2D among ethnic minorities were explained by higher levels of CRP.

## Participants and Methods

### Population

We used baseline data from the HELIUS study, collected between 2011 and 2015. HELIUS is a multi-ethnic cohort among six ethnic groups living in Amsterdam^18,19^. In brief, participants were randomly sampled from the municipality registry, stratified by ethnicity. Data were collected among 24,789 participants; questionnaires, physical examinations and biological samples were obtained^19^. CRP levels were measured in random subsamples of 1000 participants from each ethnic group (total n = 6000), who had complete data on cardiovascular measurements and stored blood samples available. In two participants, CRP could not be determined due to too little material. Additionally, participants who had T2D at baseline were excluded. Prevalent T2D was defined by self-reported physician diagnosis and/or measured fasting plasma glucose levels of ≥7.0 mmol.l^−1^ and/or anti-diabetic medication use. Finally, a total of 559 participants were lost to follow-up (7.4–11.5% per ethnic group). Therefore, 837 participants of Dutch, 712 of South-Asian Surinamese, 797 of African Surinamese, 804 of Ghanaian, 817 of Turkish and 778 of Moroccan ethnicity were included in the current study. HELIUS was approved by the Ethics Committee of the Amsterdam Medical Center (MREC 10/100# 17.10.1729). Informed consent was obtained from all individual participants included in the study.

### Measurements

Ethnicity was defined by the individual’s country of birth combined with the parental countries of birth^20^. Non-Dutch ethnic origin was assigned to participants born abroad with at least one parent born abroad or born in the Netherlands with both parents born abroad. The Surinamese group was further classified according to self-reported ethnic origin^19^.

Information on packyears of smoking and physical activity were determined from the questionnaire. Packyears was calculated by multiplying the number of packs (containing 20 cigarettes) smoked a day by the number of years. Smoking cigars and pipe tobacco were also included by calculating the equivalent rates of tobacco. The physical activity score was derived by the SQUASH questionnaire^21^, results were converted to minutes per week and multiplied by the metabolic equivalent (MET) intensity score. The total score was subdivided in quartiles, because of its skewed distribution. Body Mass Index (BMI) was determined by dividing measured body weight (kg) by height squared (m^2^). Weight and height were measured in barefoot participants wearing light clothes only. Waist circumference (WC) was measured using a tape measure at the level midway between the lowest rib margin and the iliac crest. All anthropometric measures were taken in duplicate and the mean was used in the analyses. Family history of diabetes was derived from the questionnaire.

High-sensitive CRP (hs-CRP) levels were measured in heparin plasma by a particle enhanced immunoturbidimetric assay. Human CRP agglutinates with latex particles were coated with monoclonal anti-CRP antibodies. The aggregates were determined turbidimetrically with a Cobas 702c analyser (Roche Diagnostics, Mannheim, Germany). In 624 participants, the CRP-value was below the detection limit (<0.3 mg/L) and replaced by a value of 0.15 mg/L. Because the distribution of CRP levels was skewed, we used a log-10 transformation and divided the derived values by the standard deviation before analysis.

### Incident diabetes

Incident T2D cases were identified through record linkage with two health care registrations. First, HELIUS data were linked to reimbursement data from the Achmea insurance company (Achmea Health Database) which were registered from January 1, 2010 until April 30, 2016. 15,461 participants could be linked to the Achmea data (77.7% of 19,895 with linkage consent and available Citizen Service Number). A trusted third party was used for linking the data by Citizen Service Number. To define incident T2D, we considered the use of glucose lowering medication (ATC codes A10).

Second, HELIUS data was linked to Vektis data registered from January 1, 2011 until December 31, 2017. Vektis collects insurance data of all insurance companies in the Netherlands. This additional step was undertaken to identify participants who were not insured with Achmea, increase follow-up duration, and to identify participants who received multidisciplinary care for their T2D. Linkage was done by probabilistic linkage^22^ based on date of birth, sex, and postal code. Records were matched if the probability of being a match was at least 95%. Records with the highest probability of being a match were selected. In case of a similar probabilities, diabetes statuses were inspected. In case of equal statuses this status was selected, otherwise T2D status was set to missing. In total, 18,425 participants were linked with Vektis data (89% of 20,681 with linkage consent). To define incident T2D, we considered either receiving multidisciplinary care based on care performance codes or use of pharmacological medication for diabetes registered by codes FKG_D2a and FKG_D2b.

In total, we were able to link 96.4% of all the HELIUS participants who had provided permission for data linkage (n = 19,932 of 20,681) with at least one of two data sources. Finally, we defined incident T2D as the first registration of one of the considered codes in either the Achmea database or the Vektis database. We checked whether participants had a registration with one of the considered codes before their inclusion in HELIUS, this was not the case for any of the participants.

### Statistical analyses

We first verified whether the association between CRP and T2D incidence differed by sex in models adjusted for age and ethnicity (data not shown). We did not stratify the analyses by sex, as no interaction was found.

Baseline characteristics of the participants were presented, and incidence density of T2D per 1000 person-years was determined by dividing the number of incident T2D cases by the follow-up duration. Follow-up duration was determined from inclusion date within HELIUS until loss to follow-up, latest moment of data linkage, or the moment that a participant developed T2D, and rounded up to whole years. Differences in CRP levels between ethnic minority populations and the Dutch were additionally investigated using multiple linear regression.

The association of CRP levels with incident T2D was investigated using Cox regression and presented as hazard ratios per SD increase with their corresponding 95% confidence intervals (HR [95% CI]). We investigated whether the association between CRP and T2D differed by ethnic origin by adding multiplicative interaction terms between CRP and ethnic groups in our models.

Finally, we analysed in each model whether ethnic differences in T2D were (partly) explained by CRP. This was done by estimating the association between ethnicity and T2D and comparing HRs before and after adjustment for CRP levels in each of the models.

As sensitivity analysis we repeated all analyses for participants with CRP levels above 10 mg/L (n = 181) excluded from the analyses as this can indicate an acute inflammation^23^, and our interest was in low-grade inflammation (Appendix 1). In an additional model, we adjusted for education level to be able to assess whether the association between ethnicity and T2D risk is due to the lower socio-economic status (SES) of ethnic minorities (data not shown). SES was not considered in the main analyses as confounding variable, because we believe it to partly causally mediate the association between ethnic background and T2D and ethnic differences may erroneously appear smaller. Post-hoc, family history of T2D was considered as well.

We used step-wise adjustment for confounders in our models, and adjusted for ethnicity (where needed), age, sex, physical activity, smoking, BMI and WC. This was in accordance with our conceptual model shown at http://dagitty.net/m1n0V9C.

All analyses were conducted in R version 3.5.1. P-values < 0.05 were considered as statistically significant.

## Results

The amount of women included ranged from 52.2% among South-Asian Surinamese to 66.1% among Moroccans (Table 1). The mean age ranged from 38.9 years in the Moroccan origin group to 47.2 years in the African Surinamese origin group. Ethnic minority origin populations had relatively higher levels of obesity measures than the Dutch origin population. During a median follow-up of 4.0 years [IQR 3.0; 4.0], 124 participants developed T2D. The incidence density per 1000 Person-Years varied from 3.0 among Dutch to 11.7 among South-Asian Surinamese participants.

**Table 1:** Baseline characteristics by ethnic group.

	Dutch	South-Asian Surinamese	African Surinamese	Ghanaian	Turkish	Moroccan
n	837	712	797	804	817	778
% Men (n)	47.3 (396)	47.8 (340)	39.6 (316)	40.3 (324)	43.1 (352)	33.9 (264)
Age (years)	45.5 (13.9)	43.8 (13.3)	47.2 (12.4)	44.4 (11.3)	39.6 (11.7)	38.9 (12.6)
% Smoking (n)	25.9 (217)	27.4 (195)	32.7 (261)	5.2 (42)	33.9 (277)	13.1 (102)
Physical activity (met/week)	7830 (5652; 10320)	6660 (4301; 9405)	7270 (4560; 11520)	5956 (3099; 10593)	5070 (2520; 8400)	5575 (3000; 8705)
BMI (kg/m^2^)	24.5 (3.8)	25.6 (4.2)	27.2 (5.1)	28.3 (4.5)	27.9 (5.1)	27.1 (5.0)
WC (cm)	88.9 (12.1)	89.7 (11.3)	91.0 (12.5)	92.7 (11.5)	92.4 (13.0)	90.9 (12.7)
% Family history diabetes (n)						
Missing data	8.4 (70)	11.1 (79)	13.0 (104)	16.0 (129)	11.1 (91)	14.1 (110)
Yes	17.2 (144)	52.2 (372)	38.0 (303)	17.2 (138)	41.4 (338)	45.8 (356)
No	65.5 (548)	30.2 (215)	37.9 (302)	44.2 (355)	39.3 (321)	35.1 (273)
Unknown	9.0 (75)	6.5 (46)	11.0 (88)	22.6 (182)	8.2 (67)	5.0 (39)
Incidence density T2D per 1000 Person Years (n cases)	3.0 (10)	11.7 (33)	6.8 (22)	9.0 (28)	6.6 (19)	5.0 (12)
CRP (mg/L)	0.8 (0.4; 1.9)	1.6 (0.7; 3.3)	1.4 (0.6; 3.3)	1.0 (0.5; 2.3)	1.4 (0.6; 3.4)	1.5 (0.7; 3.6)

Data are mean (SD), median (IQR) or n (%). BMI = Body Mass Index; WC = Waist Circumference.

**Figure 1:**
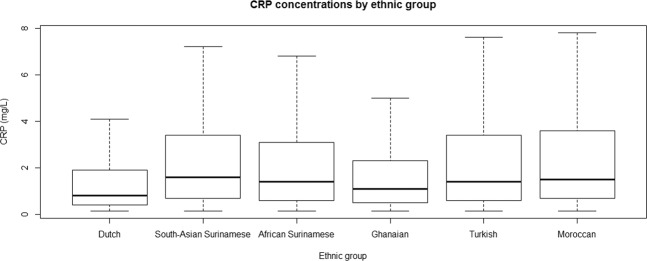
Distribution of CRP (mg/L) by ethnic group.

**Figure 2:**
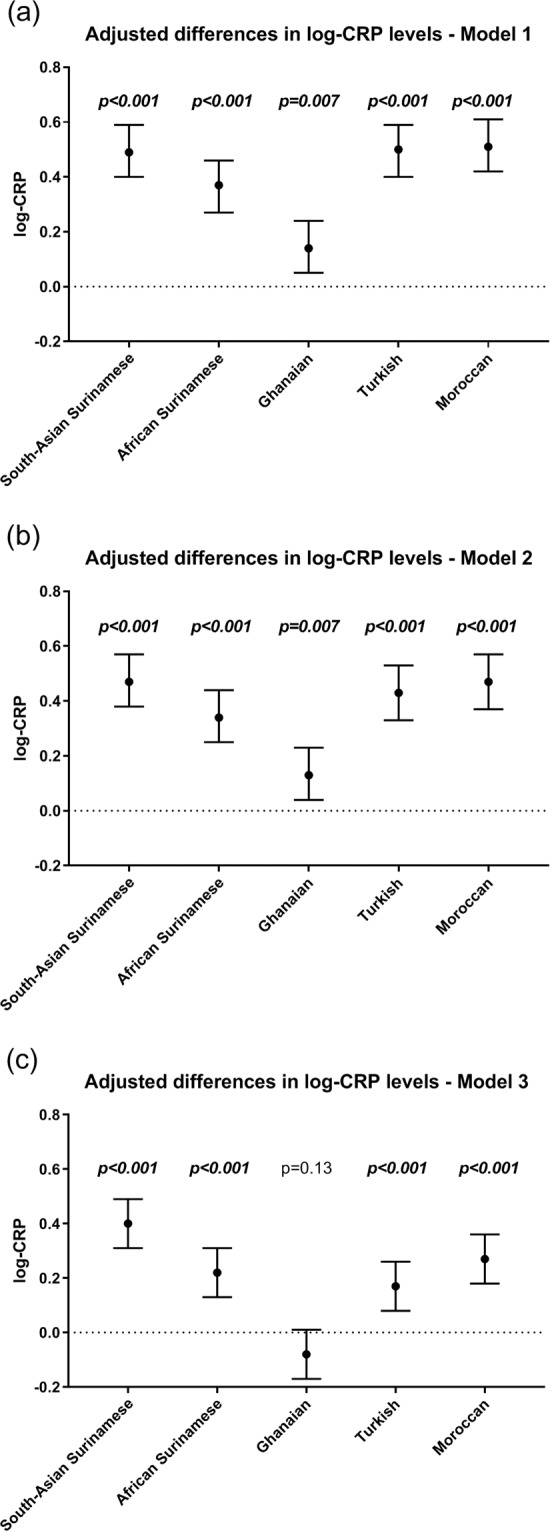
Adjusted differences in log-CRP levels (standardized beta’s) between ethnic minority groups and Dutch (reference). (**a**) Model 1 was adjusted for sex and age. (**b**) Model 2 was adjusted for sex, age, smoking and physical activity. (**c**) Model 3 was adjusted for sex, age, smoking, physical activity, BMI and waist circumference.

Median baseline CRP levels were lowest among the Dutch and highest among South-Asian Surinamese (Fig. 1). CRP levels adjusted for age, and further adjusted for smoking and physical activity, were statistically significantly higher among all ethnic minority groups as compared with Dutch participants (Fig. 2 and Appendix 2). The ethnic differences remained after additional adjustment for BMI and WC in all but Ghanaian participants.

**Figure 3:**
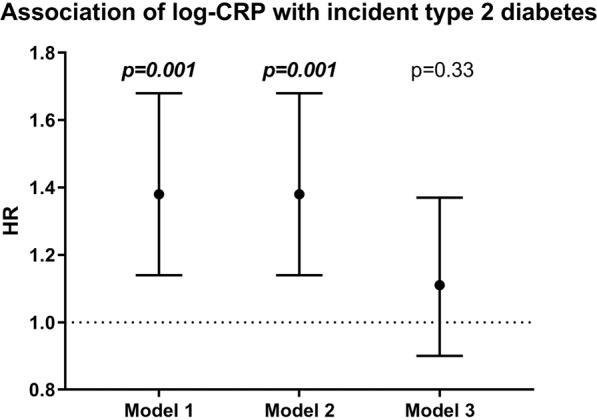
Association of log-CRP with incident type 2 diabetes in the total population. HR = hazard ratio per standard deviation increase in the 10 log transformed CRP concentration. Model 1 was adjusted for ethnicity, age and sex; model 2 additionally for smoking and physical activity; model 3 additionally for BMI and waist circumference.

We did not find statistical evidence for associations between CRP and T2D being modified by ethnicity (Appendix 3). Because of this, analyses were combined for all ethnic groups, with adjustment for ethnicity (Fig. 3 and Appendix 4). Higher levels of CRP were associated with T2D incidence in models adjusted for age, smoking and physical activity. The association was attenuated and no longer statistically significant when further adjusted for BMI and WC.

**Figure 4:**
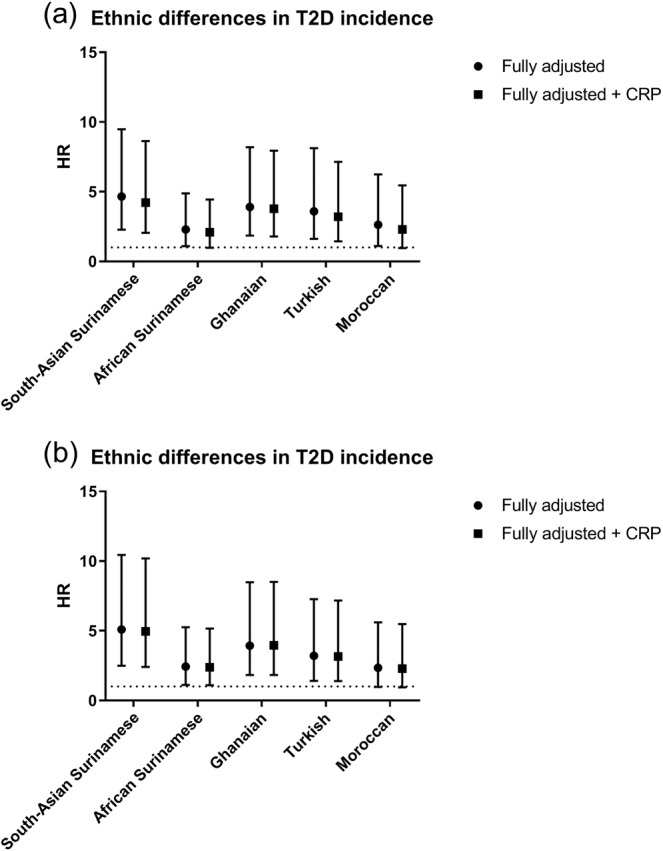
Ethnic differences in T2D incidence. T2D incidence in ethnic minorities was compared to T2D incidence in Dutch (reference). HR = hazard ratio, 95% CI = 95% confidence interval. (**a**) The fully adjusted model was adjusted for sex, age, smoking and physical activity. (**b**) The model was adjusted for sex, age, smoking, physical activity and additionally for adiposity levels, which included BMI and waist circumference.

The incidence of T2D was significantly higher among South-Asian Surinamese, African Surinamese, Ghanaian, Turkish and Moroccan as compared with Dutch (Fig. 4 and Appendix 6). A limited part of the ethnic differences in T2D incidence was explained by CRP in the models unadjusted for BMI and WC in South-Asian Surinamese, African Surinamese, Turkish and Moroccan participants, but not in Ghanaian participants. In the models including BMI and WC, CRP did not further explain ethnic differences in T2D prevalence or incidence.

All observed results remained similar after exclusion of participants who had CRP levels that might indicate acute inflammation (>10.0 mg/L; Appendix 1). The observed findings also remained similar in additional analyses with adjustment for SES and family history of diabetes (data not shown).

## Discussion

Our study shows that CRP levels are higher among ethnic minority participants of South-Asian Surinamese, African Surinamese, Turkish and Moroccan origin than Dutch participants, but similar among Ghanaians, suggesting ethnic differences in low grade inflammation. Furthermore, positive associations between CRP and T2D are observed, without evidence for different associations between CRP and T2D across ethnic groups. A very small part of the higher risk for T2D among ethnic minorities explained by higher levels of CRP. Analyses with further adjustment for BMI and WC suggest that this may be because CRP levels partly differ due to differences in adiposity levels among ethnic groups.

### Limitations

CRP was used as a marker of inflammation, but does not necessarily reflect the degree of inflammation of individual tissues. CRP is mainly produced in the liver and mainly induced by IL-6 which mainly marks inflammation of adipose tissue. Therefore, the relative contribution of liver and adipose tissue to circulating CRP is disproportionally compared to other tissues^24^. Moreover, CRP is only one marker of inflammation and other markers may provide different insights. This may prove problematic in case various markers are expressed differently across ethnic groups. However, there is no evidence available to support this.

Incident T2D was determined from insurance data. Therefore, only those participants who received diabetes care were registered as T2D cases. We will have missed participants who despite having developed T2D were not diagnosed with T2D, for instance because they did not visit their GP during the period of follow-up or due to registration issues. Results should therefore be interpreted with care, especially since screening rates for T2D between ethnic groups may differ due to differences in awareness of T2D risk across ethnic groups; awareness rates were previously shown to be higher among ethnic minority populations compared to the Dutch^2^. Moreover, we used probabilistic data linkage to link to the Vektis database, record pairs may have falsely been identified as match (i.e. belonging to the same individual). However, we chose a relatively high threshold to be sure that linked data was of high enough quality^25^. A potential downside of setting such a high threshold is that we could have missed some true matches, particularly in groups where partially identifying variables are less distinctive, e.g. date of birth in Moroccans. However, due to the large number of observations in both datasets, we considered this to be less important. Moreover, our linkage methods showed a 98% overlap in T2D statuses among participants identified in both databases, and analyses with prevalent T2D confirmed our longitudinal results (Appendices 6 and 7).

### Discussion of key findings

Earlier studies described higher CRP levels among South-Asian compared to European populations, and Moroccan and Turkish compared to Dutch populations^12,15–17^, and similar levels of CRP among Ghanaian compared to Dutch populations^14^. Our study confirmed these results and expanded the evidence to African Surinamese populations. Ethnic differences were partly explained by ethnic differences in adiposity levels. Adipocytes excrete some CRP, but adipocytes mainly increase the amount of CRP by indirect stimulation by IL-6^26^. However, higher CRP levels remained in our study among South-Asian Surinamese, African Surinamese, Turkish and Moroccan participants compared to Dutch participants after adjustment for estimates of adiposity. This is in line with other studies that showed that differences in BMI levels are not likely to fully account for the ethnic differences in CRP levels^9,10^. However, not all previous studies took the potential ethnic differences in body composition into account. BMI may explain different proportions of CRP levels within various ethnic groups, due to different correlates with body fat distribution^27^. To account for these differences in body fat distribution, our analyses were adjusted for WC in addition to BMI. These analyses showed that some ethnic differences remain even after adjustment for WC. As WC cannot fully replace an estimate of body fat mass and distribution by DEXA^28^, we cannot exclude the possibility that the slightly elevated adjusted levels of CRP indicating low-grade systemic inflammation in ethnic minority populations compared to the Dutch are partly due to residual confounding. However, other underlying factors may also contribute to the observed differences; factors likely to contribute are ethnic differences in underlying medical conditions, second hand smoke exposure, air pollution, other factors resulting in tissue injury or stress and heritability^4,15,29^.

It has previously been hypothesized that inflammation plays an important role in the pathogenesis of T2D, although its relative importance remains unknown^24^. Our study shows a positive association of CRP with incident T2D that is consistent with associations previously described. We did not find differing associations between CRP and T2D across ethnic groups of Dutch, South-Asian Surinamese, African Surinamese, Ghanaian, Turkish and Moroccan origin living in the Netherlands. This might be due to limited power. However it is not to be expected that, if differences in association between the ethnic groups under study do exist, these will be large. A meta-analyses showed positive associations between CRP and incident T2D in all studies among ethnic groups of North-American, European and Asian background, except for two studies among two north-American Aboriginal populations^5^, this might be a true association or due to study design. Altogether we suggest that there is limited to no evidence that the association between CRP and incident T2D differs across ethnic groups. In line with our study, associations between CRP and T2D were attenuated in studies that adjusted for either WC or waist-to-hip ratio^5^. In our study, the association between CRP and T2D was no longer statistically significant after adjustment for measures of adiposity, but this might also in part be due to limited power. The attenuation of the association between CRP and incident T2D after adjustment for adiposity, as observed in our and previous studies, may be related to the inflammatory response in which oxidative stress, ectopic lipid deposition, lipotoxicity and glucotoxicity are key factors underlying insulin resistance and loss of β-cell function^3,6,30^.

## Conclusion

This study aimed to clarify whether the increased risk for T2D among ethnic minorities could be explained by low-grade inflammation, as reflected by CRP. We showed that, although CRP levels are higher among ethnic minorities compared to Dutch, and CRP is consistently associated with T2D across ethnic groups, only a very small part of the increased risk for T2D is explained by CRP. Most of the increased risk for T2D among ethnic minorities explained by CRP are attenuated when adiposity levels are taken into account. We, therefore, conclude that reduction of low-grade inflammation among ethnic minorities may have limited effects in reducing the increased risk for T2D among ethnic minorities.

### Ethical approval

All procedures performed in studies involving human participants were in accordance with the ethical standards of the institutional and/or national research committee and with the 1964 Helsinki declaration and its later amendments or comparable ethical standards.

## Supplementary information

Supplementary Tables

## Data Availability

The HELIUS data are owned by the Academic Medical Center (AMC) in Amsterdam, The Netherlands. Any researcher can request the data by submitting a proposal to the HELIUS Executive Board as outlined at http://www.heliusstudy.nl/en/researchers/collaboration.
