# Supplementary material for: The high risk for type 2 diabetes among ethnic minority populations is not explained by low-grade inflammation
Source: Sci Rep. 2019 Dec 27;9:19871. doi: 10.1038/s41598-019-56596-4 (PMC6934847; doi:10.1038/s41598-019-56596-4)
Supplement: Supplementary file 1 — Supplementary Tables [file 41598_2019_56596_MOESM1_ESM.docx]

**The high risk for type 2 diabetes among ethnic minority populations is not explained by low-grade inflammation.**

**Mirthe Muilwijk, Max Nieuwdorp, Marieke B. Snijder, Michel H.P. Hof, Karien Stronks, Irene G.M. van Valkengoed**

**Appendix 1: Association of CRP <10mg/L with incident type 2 diabetes**

|  | HR (95% CI) | p-value |
| --- | --- | --- |
| Model 1 | 1.42 (1.14; 1.78) | ***0.002*** |
| Model 2 | 1.43 (1.14; 1.79) | ***0.002*** |
| Model 3 | 1.10 (0.87; 1.40) | 0.43 |

*HR= Hazard ratio per standard deviation increase in the 10log transformed CRP concentration. Model 1 was adjusted for ethnicity, age and sex; model 2 additionally for smoking and physical activity; model 3 additionally for BMI and waist circumference.*

**Appendix 2: Adjusted differences in log-CRP levels (standardized beta’s (sBeta)) between ethnic minority groups and Dutch (reference)**

|  | **South-Asian Surinamese** | | **African Surinamese** | | **Ghanaian** | | **Turkish** | | **Moroccan** | |
| --- | --- | --- | --- | --- | --- | --- | --- | --- | --- | --- |
|  | **sBeta (95% CI)** | **p-value** | **sBeta (95% CI)** | **p-value** | **sBeta (95% CI)** | **p-value** | **sBeta (95% CI)** | **p-value** | **sBeta (95% CI)** | **p-value** |
| **Model 1** | 0.49 (0.40; 0.59) | ***<0.001*** | 0.37 (0.27; 0.46) | ***<0.001*** | 0.14 (0.05; 0.24) | ***0.007*** | 0.50 (0.40; 0.59) | ***<0.001*** | 0.51 (0.42; 0.61) | ***<0.001*** |
| **Model 2** | 0.47 (0.38; 0.57) | ***<0.001*** | 0.34 (0.25; 0.44) | ***<0.001*** | 0.13 (0.04; 0.23) | ***0.007*** | 0.44 (0.34; 0.54) | ***<0.001*** | 0.49 (0.39; 0.59) | ***<0.001*** |
| **Model 3** | 0.40 (0.31; 0.49) | ***<0.001*** | 0.22 (0.13; 0.31) | ***<0.001*** | -0.07 (-0.16; 0.02) | 0.13 | 0.19 (0.10; 0.28) | ***<0.001*** | 0.30 (0.21; 0.39) | ***<0.001*** |

*Model 1 was adjusted for age and sex; model 2 additionally for smoking and physical activity; model 3 additionally for BMI and waist circumference.*

**Appendix 3: Association of CRP with incident type 2 diabetes stratified by ethnicity**

|  | Dutch |  | South-Asian Surinamese | | African Surinamese | | Ghanaian | | Turkish | | Moroccan | |
| --- | --- | --- | --- | --- | --- | --- | --- | --- | --- | --- | --- | --- |
|  | HR (95% CI) | p-value | HR (95% CI) | p-value | HR (95% CI) | p-value | HR (95% CI) | p-value | HR (95% CI) | p-value | HR (95% CI) | p-value |
| Model 1 | 1.58 (0.83; 3.01) | 0.16 | 1.31 (0.87; 1.98) | 0.20 | 1.40 (0.89; 2.21) | 0.14 | 1.15 (0.78; 1.69) | 0.49 | 2.13 (1.20; 3.78) | ***0.01*** | 1.32 (0.76; 2.42) | 0.38 |
| Model 2 | 1.57 (0.79; 3.11) | 0.20 | 1.30 (0.86; 1.98) | 0.21 | 1.41 (0.89; 2.24) | 0.14 | 1.15 (0.78; 1.68) | 0.49 | 2.10 (1.17; 3.76) | ***0.01*** | 1.33 (0.72; 2.45) | 0.37 |
| Model 3 | 1.27 (0.58; 2.75) | 0.55 | 1.14 (0.74; 1.77) | 0.55 | 1.18 (0.70; 1.99) | 0.53 | 0.96 (0.63; 1.45) | 0.83 | 1.44 (0.74; 2.81) | 0.28 | 1.07 (0.56; 2.06) | 0.83 |

*HR= hazard ratio per standard deviation increase in the 10log transformed CRP concentration. Model 1 was adjusted for age and sex; model 2 additionally for smoking and physical activity; model 3 additionally for BMI and waist circumference. None of the p-values for interactions between CRP and ethnicity with type 2 diabetes incidence as the outcome was statistically significant.*

**Appendix 4: Association of log-CRP with incident type 2 diabetes in the total population**

|  | HR (95% CI) | p-value |
| --- | --- | --- |
| Model 1 | 1.38 (1.14; 1.68) | ***0.001*** |
| Model 2 | 1.38 (1.14; 1.68) | ***0.001*** |
| Model 3 | 1.11 (0.90; 1.37) | 0.33 |

*HR= hazard ratio per standard deviation increase in the 10log transformed CRP concentration. Model 1 was adjusted for ethnicity, age and sex; model 2 additionally for smoking and physical activity; model 3 additionally for BMI and waist circumference.*

**Appendix 5:** **Ethnic differences in type 2 diabetes incidence, additionally adjusted for CRP and/or adiposity measures**

|  | **Dutch** | **South-Asian Surinamese** | **African Surinamese** | **Ghanaian** | **Turkish** | **Moroccan** |
| --- | --- | --- | --- | --- | --- | --- |
|  | **HR** | **HR (95% CI)** | **HR (95% CI)** | **HR (95% CI)** | **HR (95% CI)** | **HR (95% CI)** |
| **Fully adjusted model** | 1.00 | 4.65 (2.28; 9.48) | 2.30 (1.09; 4.88) | 3.90 (1.86; 8.20) | 3.59 (1.62; 7.96) | 2.63 (1.11; 6.24) |
| **Fully adjusted model + CRP** | 1.00 | 4.22 (2.06; 8.63) | 2.09 (0.98; 4.44) | 3.78 (1.80; 7.95) | 3.21 (1.44; 7.14) | 2.29 (0.96; 5.45) |
| **Fully adjusted model + adiposity** | 1.00 | 5.10 (2.49; 10.45) | 2.43 (1.12; 5.25) | 3.93 (1.83; 8.48) | 3.21 (1.41; 7.27) | 2.34 (0.97; 5.61) |
| **Fully adjusted model + adiposity + CRP** | 1.00 | 4.96 (2.41; 10.19) | 2.38 (1.10; 5.16) | 3.95 (1.83; 8.51) | 3.16 (1.39; 7.17) | 2.28 (0.95; 5.48) |

*HR = hazard ratio, 95% CI = 95% confidence interval. The fully adjusted model was adjusted for age, sex smoking and physical activity; adiposity included BMI and waist circumference.*

**Appendix 6: Association of CRP with prevalent type 2 diabetes**

|  | OR (95% CI) | p-value |
| --- | --- | --- |
| Model 1 | 1.20 (1.09; 1.32) | ***<0.001*** |
| Model 2 | 1.19 (1.08; 1.31) | ***<0.001*** |
| Model 3 | 0.96 (0.87; 1.07) | 0.48 |

*OR= odds ratio per standard deviation increase in the 10log transformed CRP concentration. Model 1 was adjusted for ethnicity, age and sex; model 2 additionally for smoking and physical activity; model 3 additionally for BMI and waist circumference.*

**Appendix 7: Ethnic differences in type 2 diabetes prevalence adjusted for CRP**

|  | **Dutch** | **South-Asian Surinamese** | **African Surinamese** | **Ghanaian** | **Turkish** | **Moroccan** |
| --- | --- | --- | --- | --- | --- | --- |
|  | **OR** | **OR (95% CI)** | **OR (95% CI)** | **OR (95% CI)** | **OR (95% CI)** | **OR (95% CI)** |
| **Fully adjusted model** | 1.00 | 8.03 (5.53; 11.97) | 3.17 (2.13; 4.80) | 5.30 (3.58; 8.02) | 5.45 (3.61; 8.39) | 6.60 (4.42; 10.06) |
| **Fully adjusted model + CRP** | 1.00 | 7.73 (5.32; 11.54) | 3.04 (2.05; 4.61) | 5.29 (3.57; 8.02) | 5.14 (3.39; 7.92) | 6.23 (4.17; 9.52) |
| **Fully adjusted model + adiposity** | 1.00 | 9.08 (6.18; 13.67) | 3.52 (2.33; 5.42) | 5.86 (3.89; 9.03) | 5.03 (3.26; 7.89) | 6.28 (4.15; 9.70) |
| **Fully adjusted model + adiposity + CRP** | 1.00 | 9.14 (6.22; 13.78) | 3.54 (2.34; 5.45) | 5.83 (3.87; 8.98) | 5.05 (3.27; 7.92) | 6.32 (4.17; 9.76) |

*OR= odds ratio, 95% CI = 95% confidence interval. Model 1 was adjusted for age, sex smoking and physical activity; model 2 additionally for CRP; model 3 additionally for BMI and waist circumference.*
